# IgG4-Related Disease With Testicular Involvement: A Case Report and Review of Literature

**DOI:** 10.3389/fimmu.2021.717902

**Published:** 2021-08-10

**Authors:** Gang Wang, Ning Zhuo, Xiaowen Luo, Feng Tian, Zhenhua Wen, Jingyang Li

**Affiliations:** ^1^Department of Rheumatology and Immunology, Zhuzhou Hospital Affiliated to Xiangya Medical College, Central South University, Zhuzhou, China; ^2^Department of Nephrology, The Second Xiangya Hospital, Central South University, Changsha, China

**Keywords:** IgG4-related disease, testis, clinical feature, treatment, glucocorticoids

## Abstract

Immunoglobulin G4-related disease (IgG4-RD) is an autoimmune inflammatory disease characterized by infiltration of IgG4+ plasma cells that can simulate a tumor manifesting as a tumor-like mass. This disease involves the pancreas, biliary tract, kidneys, salivary glands, lymph nodes, aorta, and retroperitoneum amongst other organs. However, testicular involvement is a rare entity in this disease. The treatment of testicular involvement in IgG4-RD is currently controversial. We present the case of a 65-year-old man with swelling and pain in his right scrotum three months ago. On examination, a mobile mass of approximately 2 cm in diameter was found in the right scrotum. Serological tests showed elevated levels of IgG4 and negative for tumor markers. Enhanced computed tomography of the scrotum showed a nodular hyperdense shadow with a diameter of approximately 23 mm on the right epididymis. Pathological biopsy of the right epididymis showed infiltration of plasma cells, lymphocytes, and a few neutrophils. IgG4+ plasma cells stained positive, with an IgG4/IgG ratio of more than 40% and more than 30 IgG4+ plasma cells per high-power field. A diagnosis of IgG4-RD involving the testicles was made. Prednisone 30 mg/d was given for three weeks. No scrotum swelling or pain was observed at the follow-up after six months. IgG4-related disease should be considered whenever a mass-like lesion with typical histomorphologic features involving multiple organs/anatomical sites is encountered. The testicles are an important male reproductive organ, especially for young male patients with fertility requirements. For patients with IgG4-RD testicular involvement, surgical or medical treatment requires further study.

## Introduction

IgG4-RD is an autoimmune inflammatory disease characterized by infiltration of IgG4+ plasma cells that can simulate a tumor manifesting as a tumor-like mass (1). Also, IgG4-RD is a systemic disease that involves the pancreas, biliary tract, kidneys, salivary glands, lymph nodes, aorta, and retroperitoneum amongst other organs (2). However testicular involvement is a rare entity in this disease. Until now, IgG4-RD testicular involvement has been described mainly in the form of case reports, with a lack of summary studies. Since the testis is a rarely affected entity in IgG4-RD and its clinical features are poorly understood, the purpose of this article is to raise awareness of the disease, as well as to present problems and phenomena that may need to be addressed in the future.

## Case Presentation

**Figure 1:**
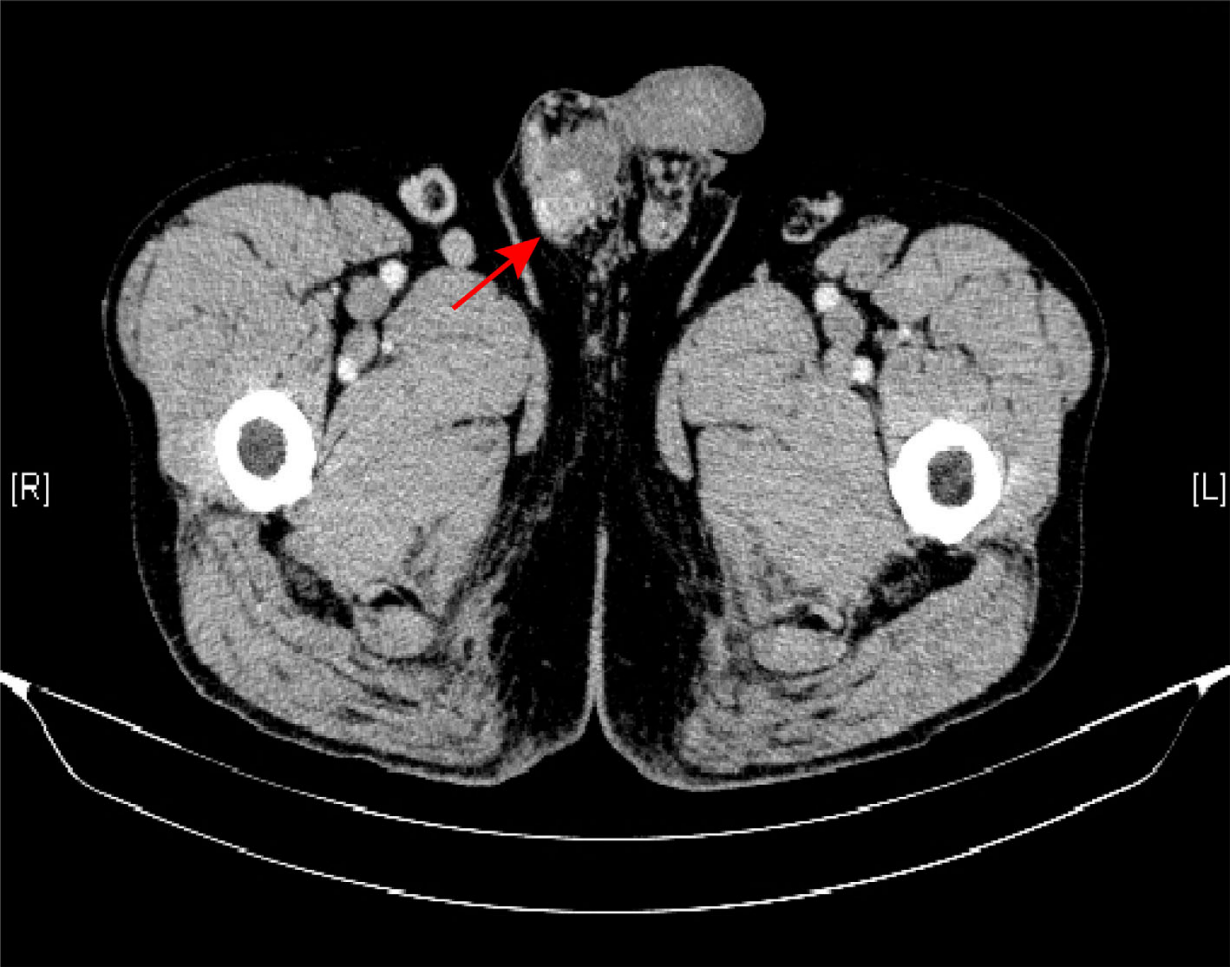
Enhanced computed tomography of the scrotum showed a nodular hyperdense shadow in the right epididymis (arrow).

**Figure 2:**
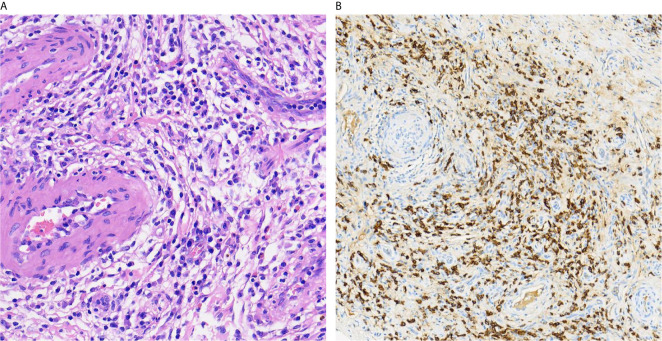
Pathological biopsy of the right epididymis showed infiltration of plasma cells, lymphocytes, and a few neutrophils with occlusive vasculitis changes in the tissue (HE, ×40) **(A)**. Immunostains showed a markedly increased number of IgG4+ plasma cells as well as an increased IgG4+/IgG+ ratio (IgG4 immunostain, ×40) **(B)**.

**Figure 3:**
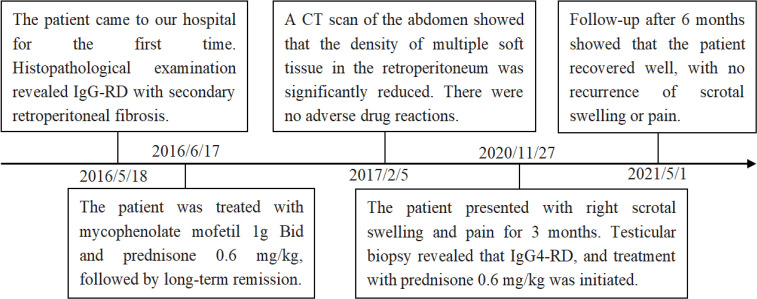
Timeline of diagnosis, treatment, and prognosis in this case.

The patient, a 65-year-old man, presented with swelling and pain in his right scrotum three months ago. He was first admitted to our hospital 5 years ago with a cough and fever. The CT scan of the abdomen showed multiple soft tissue densities behind the peritoneum and the serum IgG4 3200 mg/dL. The patient was definitively diagnosed with IgG4-RD as well as secondary retroperitoneal fibrosis by the gold standard of histopathology. He was in long-term remission after regular treatment with mycophenolate mofetil (1g Bid) and prednisone (0.6 mg/kg), followed by six months, four years, and a recent abdominal CT scan showed no progression of retroperitoneal fibrosis. He had no previous history of pulmonary or urinary tuberculosis and no significant family history. On examination, a mobile mass of approximately 2 cm in diameter was found in the right scrotum. The mass was hard and tender. No other positive signs were found. Laboratory tests showed erythrocyte sedimentation rate 69 mm/h (reference range 0-15 mm/h), C-reactive protein 29.9 mg/L (reference range 0-10 mg/L), complement C3 0.58 g/L (reference range 0.9-1.8 g/L), complement C4 0.03 g/L (reference range 0.1-0.4 g/L). Serological examination showed elevated IgG4 levels to 2070 mg/dL (reference range 6-130 mg/dL). Negative for prostate-specific antigen (PSA), tumor markers, and tuberculosis antibodies. Scrotal ultrasound demonstrating enlargement of the right testicle. Enhanced computed tomography of the scrotum showed a nodular hyperdense shadow with a diameter of approximately 23 mm on the right epididymis, with significant heterogeneous enhancement on the enhanced scan (**Figure 1**). After ruling out testicular tuberculosis and hydrocele due to retroperitoneal fibrosis secondary to IgG4-RD, given the potential risk of malignancy, he underwent a pathological biopsy by puncture of the right epididymis. Pathological biopsy of the right epididymis showed infiltration of plasma cells, lymphocytes, and a few neutrophils with occlusive vasculitis changes in the tissue (**Figure 2A**). IgG4+ plasma cells stained positive, with an IgG4/IgG ratio of more than 40% and more than 30 IgG4+ plasma cells per high-power field (**Figure 2B**). A diagnosis of IgG4-RD involving the testicles was made. Prednisone 30 mg/d was given for three weeks. No scrotum swelling or pain was observed at the follow-up after six months. The timeline of diagnosis, treatment, and prognosis for this case is shown in **Figure 3**.

## Methods

We performed a literature review of published articles in English on IgG4-RD involving the testis. We searched electronic databases (Pubmed, Embase, Cochrane Library) using the following keywords: “epididymis”, “paratestis”, “testis”, “testicular”, “scrotum”, “scrotal”, “intrascrotal”, “spermatic cord, “paratesticular fibrous pseudotumor”, “paratesticular pseudotumor”, “Epididymo-orchitis”, “Immunoglobulin G4”, “IgG4”. It is worth noting that the literature was searched by two researchers, and a third researcher made the final judgment when there was disagreement. We also reviewed the articles cited in the literature to ensure the accuracy of the retrieved content. Finally, 14 articles were included in the study (**Table 1**).

**Table 1:** Clinical characteristics of 18 patients with testicular involvement of IgG4-related diseases.

Authors	Age/years	Symptoms	Duration/months			Medical history	Serum IgG4 level (mg/dL)			Treatment	Outcomes
Garber et al. (2)	36	Small, painless right scrotal mass		ND	No			166.8	Surgical excision of the mass along with a vasectomy		Recovered
Shams et al. (3)	55	Pain and swelling in the right scrotal region		5 days	Diabetes			467	Right-sided orchidectomy; Broad spectrum antibiotics		Recovered
Mochizuki et al. (4)	74	Left-sided scrotal mass and swelling		5	Appendectomy; Prostatic cancer			109 (After Surgery)	Left inguinal orchiectomy		ND
ChangChien et al. (5)	41	Painless right intrascrotal, paratesticular mass		36	No			ND	Right orchiectomy		Recovered
Cho et al. (6)	46	Swelling in his right scrotum		1	ND			>340	Right hydrocelectomy; Prednisolone; Methotrexate		Recovered
Tokura et al. (7)	72	Painless right scrotal swelling		ND	Left hydronephrosis; Retroperitoneal fibrosis			550	Right high orchiectomy; Prednisolone		Recovered
Lal et al. (8)	35	Pain in the right hemiscrotum; Non tender hard nodule in the right testis		1	ND			Normal range	High inguinal orchiectomy		ND
Kim et al. (9)	46	Enlarged, hard mass in the right hemiscrotum		ND	Retroperitoneal fibrosis			7660	Oral steroid		Recovered
Migita et al. (10)	74	Left paratesticular mass		ND	Bladder cancer; Radical cystectomy			505	Steroid treatment; Left semicastration		Recovered
Karashima et al. (11)	33	Swelling and mass in the left scrotum		3	Wells syndrome; Periproctal abscess			31.8	Left radical orchidectomy; Oral dexamethasone sodium phosphate		Recovered
Dieckmann et al. (12)	28	Right-sided painless scrotal mass		48	No			ND	Local excision		Recovered
	19	Right-sided painless scrotal mass		6	Tonsillectomy			ND	Local excision		Recovered
de Buy Wenniger et al. (13)	64	Scrotal pain; Swelling and pain in the right testicle		6	IgG4-related pancreaticobiliary disease			ND	Oral antibiotics; Orchidectomy		ND
Hart et al. (14)	67	Painless right scrotal mass		3	Diabetes mellitus; Autoimmune pancreatitis; Retroperitoneal fibrosis; Left hydronephrosis			ND	Right inguinal radical orchiectomy; Oral prednisone		Recovered
Bösmüller et al. (15)	23	Testicular masses on both sides		ND	No			ND	Local excision		ND
	52	Rightsided, palpable paratesticular nodules		ND	No			ND	Local excision		ND
	25	Right testicular mass with swelling and pain		Several weeks	No			ND	Local excision		ND
This Case	65	Pain and swelling in the right scrotal region		3	Retroperitoneal fibrosis			2070	Oral prednisone		Recovered

ND, Not described.

## Discussion

IgG4-RD is a chronic progressive autoimmune disease characterized by elevated serum IgG4 levels as well as IgG4+ plasma cell infiltration of organs (1). The pathogenesis of IgG4-RD is still unclear, but the main view is that autoimmunity and infection are potential immune triggers for IgG4-RD, leading to overexpression of cytokines represented by type 2 helper T (Th2) cells and activation of regulatory T (Treg) cells causing massive infiltration of inflammatory cells leading to organ damage (16). IgG4-RD can involve multiple organs throughout the body, and the testis, a rare area of involvement, may also present with mass forming lesions.

A total of 18 patients with a median age of 46 years and a mean age of 47.5 years were included in the study. There is a bimodal age distribution with a concentration of people aged 20-50 years and 70 years or older. A cohort study by Inoue et al. (17) enrolled 235 IgG4-RD patients with a median age of 67 years. Wallace et al. (18) enrolled 125 IgG4-RD patients with a mean age of 50.3 years. The above data indicate that the mean age of patients with testicular involvement is much younger. A prospective study that included 737 patients with IgG4-RD showed that the proportion of superficial organ (such as the testicles) involvement decreased with age, while the proportion of visceral organ involvement increased with age, which was more visible in male patients (19). However, the patients included in this article did not fit the above findings. In terms of clinical manifestations, intra-scrotal masses were the most frequent (13/18, 72.22%), followed by swelling (8/18, 44.44%) and pain (5/18, 27.77%). Testicular painlessness was more common than patients with pain (13 *vs* 5). Testicular painlessness may be the cause of delayed diagnosis and the prolonged course of the disease. Six patients (33.33%) had no previous underlying disease and subsequently developed IgG4-RD, while four patients (22.22%) developed retroperitoneal fibrosis before the IgG4-RD involvement of the testicles. The relationship between retroperitoneal fibrosis and testicular involvement is not clarified. We hypothesize that when retroperitoneal fibrosis developed, the patient’s immune system had been attacked and no longer provided complete protection to the testicles. In terms of treatment, 16 patients (88.88%) underwent surgical treatment including partial and radical resection; only 7 patients were treated with hormone therapy (38.88%) and 1 patient was treated with methotrexate. Twelve patients were followed up and all improved.

Swelling and pain associated with IgG4-RD in the testis should be differentiated from testicular tuberculosis, testicular tumors, and hydrocele due to retroperitoneal fibrosis secondary to IgG4-RD. The patient had no previous history of tuberculosis of the lung or urinary tract, which effectively ruled out testicular tuberculosis. Secondary retroperitoneal fibrosis of IgG4-RD involving the ureter, inferior vena cava, abdominal aorta, and its branches, leading to hydrocele testis, can be the cause of testicular enlargement. However, after the patient was regularly treated with prednisone and mycophenolate mofetil for the first time in our hospital, retroperitoneal fibrosis was long in remission, which was confirmed by multiple abdominal CTs. Histopathologic diagnosis is still the gold standard for the diagnosis of this disease. Therefore, the patient ruled out any of these causes. The fact that this disease can mimic testicular malignancy and the inexperience of surgeons with this disease lead the vast majority of patients to receive surgical treatment. For the treatment of definitively diagnosed IgG4-RD, steroid hormone therapy that includes both inductions of remission and maintenance therapy may be more beneficial in rapidly resolving the patient’s symptomatic, biochemical, and imaging abnormalities (20). The testicles are a vital reproductive organ that plays an important role in regulating growth and development and maintaining male secondary sexual characteristics. A thorough evaluation should be performed especially in young patients with fertility requirements. It is necessary to enlist the assistance of rheumatologists and pathologists in cases of diagnostic difficulties. Early diagnosis and management can prevent unnecessary orchiectomy.

Glucocorticoids are the first-line agents to induce remission in patients with IgG4-RD (20). Patients with poor response to glucocorticoids should undergo additional evaluation, including repeat biopsy procedures to verify the diagnosis (20). Immunosuppressants can be an alternative option to poor hormone efficacy. Prevention of IgG4-RD relapse is the focus of maintenance therapy. In general, the risk of relapse is highest when patients present with multiple organ involvement, elevated serum IgG4, IgE, and peripheral eosinophilia (1). Thus, the importance of long-term monitoring of patient’s serum IgG4, IgE, and peripheral eosinophils is highlighted.

**Figure 4:**
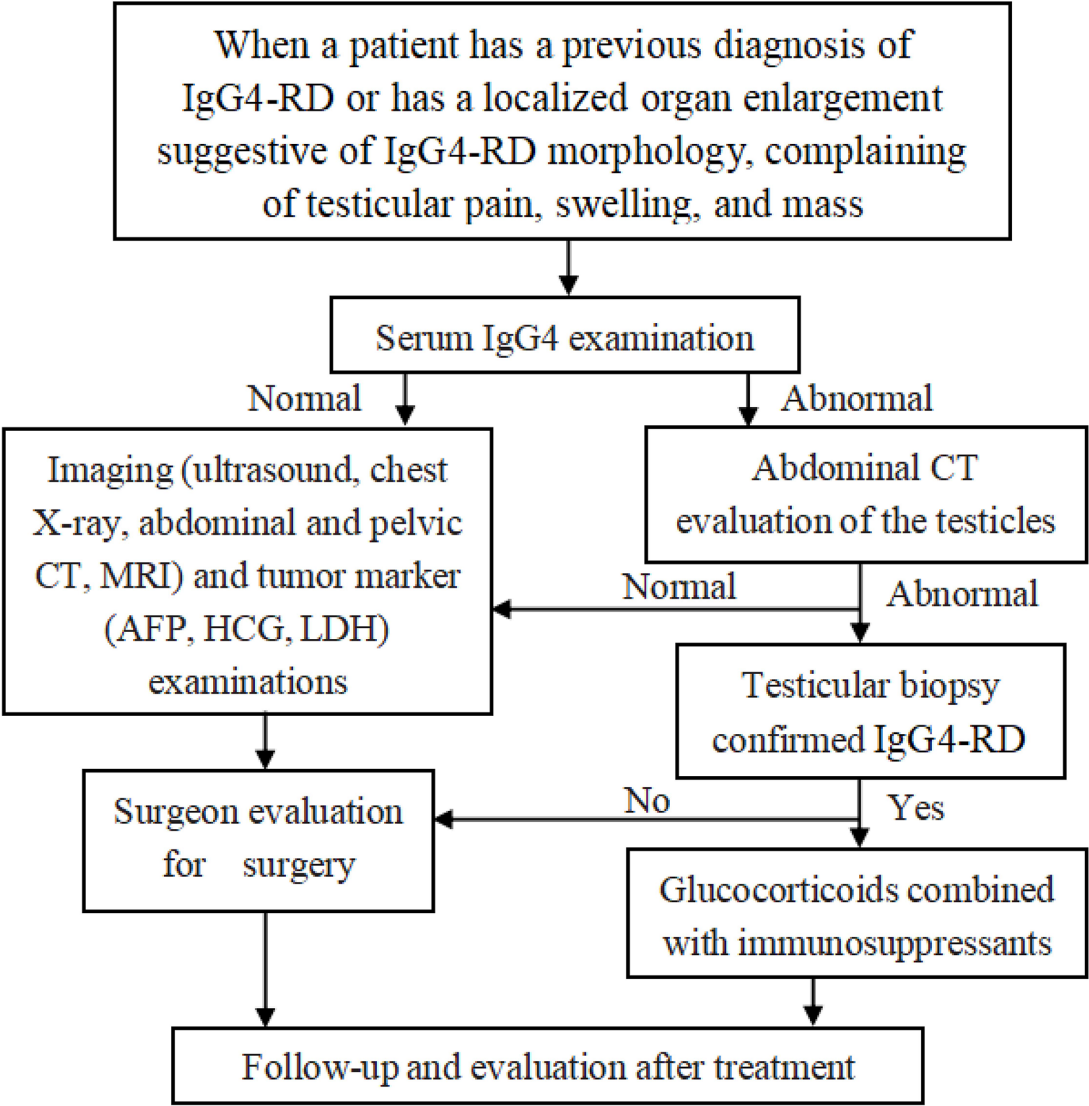
The empirically tailored, step-wise approach to the current treatment of testicular pain, swelling, and mass.

In clinical practice, in addition to careful physical examination, when a patient has a previous diagnosis of IgG4-RD or has a localized organ enlargement suggestive of IgG4-RD morphology, complaining of testicular pain, swelling, and mass, testing the serum IgG4 level is essential as it plays a vital role in the diagnosis and monitoring of IgG4-RD recurrence (6). When serum IgG4 is normal, the surgeon’s evaluation is a priority and important. The screening of imaging and tumor markers can rule out testicular tumors as early as possible. However, testicular computed tomography and testicular aspiration biopsy are preferred and important when serum IgG4 is abnormal. We recommend the involvement of rheumatologists and pathologists throughout the entire process for the early diagnosis of IgG4-RD. The disease is responsive to glucocorticoid therapy, and early diagnosis and management can prevent unnecessary orchiectomy. Notably, regular follow-up after treatment allows early detection of disease evolution (**Figure 4**). The patient also presented his point of view: when there is testicular swelling or pain, timely medical attention is crucial to avoid delay in the illness.

This study has some limitations. Testicular involvement is uncommon, thus besides presenting the case, it is difficult to provide definitive conclusions. At the same time, the data for this study is based on published literature, which suggests that there may be more cases of testicular involvement of IgG4-RD in real life than in published literature. However, based on the existing evidence, this may provide an important reference for future research and cause for concern.

Finally, we found it interesting that the right testis was significantly more involved than the left (15 *vs* 4), only Bösmüller et al. (15) reported a patient with testicular involvement on both sides. However, there are no studies that describe how the right testis predominates in this disease, and we speculate that it may be related to the local physiological anatomy. Information on the testicular endocrine function such as androgen levels and sperm production is not available in the literature. Further studies are needed to confirm whether this disease affects testicular endocrine function and by what pathway.

## Conclusion

IgG4-related disease should be considered whenever a mass-like lesion with typical histomorphologic features involving multiple organs/anatomical sites is encountered. The involvement of rheumatologists and pathologists throughout the entire process is vital for the diagnosis of IgG4-RD. Early diagnosis and management can prevent unnecessary orchiectomy.

## Data Availability Statement

The original contributions presented in the study are included in the article. Further inquiries can be directed to the corresponding author.

## Ethics Statement

Written informed consent was obtained from the individual(s) for the publication of any potentially identifiable images or data included in this article.

## Author Contributions

GW and NZ designed and wrote the paper. XL, FT, ZW, and JL reviewed and edited the manuscript. All authors contributed to the article and approved the submitted version.

## Conflict of Interest

The authors declare that the research was conducted in the absence of any commercial or financial relationships that could be construed as a potential conflict of interest.

## Publisher’s Note

All claims expressed in this article are solely those of the authors and do not necessarily represent those of their affiliated organizations, or those of the publisher, the editors and the reviewers. Any product that may be evaluated in this article, or claim that may be made by its manufacturer, is not guaranteed or endorsed by the publisher.
